# Traveling Companions Add Complexity and Hinder Performance in the Spatial Behavior of Rats

**DOI:** 10.1371/journal.pone.0146137

**Published:** 2016-01-04

**Authors:** Alex Dorfman, Kristoffer Laigaard Nielbo, David Eilam

**Affiliations:** 1 Department of Zoology, Tel-Aviv University, Ramat-Aviv, 69978, Israel; 2 Interacting Minds Center, Aarhus University, Nordre Ringgade 1, DK-8000, Aarhus C, Denmark; Brock University, CANADA

## Abstract

We sought to uncover the impact of the social environment on the spatial behavior of rats. Food-deprived rats were trained in a spatial task of collecting food items from 16 equispaced objects. Following training, they were tested, first alone and then with a similarly-trained cage-mate. It was found that the presence of another rat substantially altered the rats' spatial behavior. Lone rats collected the food items faster while traveling a shorter distance, reflecting a higher efficiency of task completion. When accompanied by a partner, however, the rats traveled together, visiting the same set of objects in each trip with one of them leading. Whether alone or with a partner, rats continued to revisit the same objects; however, more such revisits occurred with a partner. We argue that revisiting objects is not necessarily an error, since returning to past places is an important aspect of rats’ natural behavior. Revisiting an object following food depletion implies that searching for food was not the main driving force in the rats' spatial behavior. Specifically, despite food deprivation, rats were more attentive to one another than to the food. This could be adaptive, since foraging and feeding in groups is a way of poison avoidance in wild rats. Finally, the addition of a social component added complexity to the environment since the rats organized their spatial behavior in reference to one another in addition to their organization in the physical surrounding. Consequently, when tested with a partner, spatial behavior was less structured, less predictable and more chaotic.

## Introduction

Spatial behavior, which is the organization of behavior in time and space ('when' and 'where' aspects), is a main attribute of animal cognition [1]. Ever since the studies by Tolman [2,3] and O'keefe and Nadel [4], most research on spatial behavior has focused on the behavior of individuals, overlooking the possible impact of the social environment. However, social animals tend to be attracted to conspecifics and stay or travel together [5–7]. Moreover, spatial behavior in social animals needs to be organized also in reference to their social group [8]. Accordingly, the primary aim of the present study was to examine how spatial behavior of individuals is affected by the presence of a conspecific ('when' and 'where' in the context of 'with whom'). A recent study on the exploratory behavior of a dyad of rats in a large plain open field revealed that the two rats were attracted to one another, turning frequently to face one another, with one of the rats usually in the lead, virtually ignoring the physical terrain and concentrating on the social environment [9]. The present study sought to confirm the dominance of the social environment over the physical environment and the need to forage. Dyads of food-deprived rats were tested in a structured environment comprised of a large open field with 16 equispaced objects, each baited with a piece of chocolate-flavored cereal. The rats thus faced a conflict between their social preference to travel together in a structured environment [9] and their desire to obtain the food.

A propensity to socializing and sharing food among rats was demonstrated in a previous study, in which a freely-moving rat was placed in a small arena with a cage-mate held in a cage while a few pieces of chocolate were placed in another location. The free rats either preferred to release the caged rat before eating the chocolate, and thus eventually sharing it with the partner. Alternatively, the free rat first ate part of the food, leaving some for its mate which it released afterwards [10]. That study demonstrated that social factors dominate over desire for food in rats. Whereas Ben-Ami Bartal and her colleagues tested satiated rats [10], in the present study the rats were food-deprived, and thus were confronted with a conflict between socializing and competing over food. If these rats were to reflect the findings of Ben-Ami Bartal and her colleagues, they should socialize rather than compete. Alternatively, they could forage for the food independently, with each trying to consume it before its partner. In other words, the question posed in the present study was that of which factor dominates spatial behavior in food-deprived rats: preference for food or for a companion?

When engaging in routine activities, animals directly or indirectly provide information-bearing cues or signals to conspecifics and others. Such cues and signals are embedded in many examples of social learning regarding when, where, what, and how to eat [11]. It was suggested that bird roosts serve as information centers from which unsuccessful foragers can follow successful foragers to patchy, rich, but transient feeding sites [12]. Subsequent studies provided evidence for 'local enhancement'–a process in which animals travel to and feed in locations where they see others feeding [13–21]. In other words, a demonstrator incidentally attracts an observer to a specific location, leading to the observer learning. The influence of local enhancement on the selection of feeding sites by rodents has received considerable attention [22–25]. For example, the mere presence of an adult Norway rat, even an anaesthetized one, at a feeding site, results in conspecific juveniles approaching from a distance and eating at that site. Similarly, juvenile rats preferred sites where adults were feeding over sites where pups were feeding [26]. Adult rats also deposit persistent chemicals at feeding sites and on the foods they exploit [27,28], leaving scent trails as they travel between feeding sites and nests. Juvenile rats consequently prefer to explore and feed at such marked sites [25]. In line with the above-mentioned studies, laboratory rats (Wistar and Listar hooded strains) exhibit social foraging as well as other components of natural rat behavior [29]. This tendency to social feeding returns us to our question of whether the food-deprived rats in the present study would travel and feed together or compete for the available food.

While organizing behavior in time and space is a basic and vital cognitive necessity [1], the need to orient in relation to the social environment adds another level of complexity. For example, an individual rat organizes its exploration as roundtrips to a key location termed the "home base" [30]. In this case, the single rat considers only the physical environment. In contrast, an infant chimpanzee that rides its mother's back, descends for roundtrips in the environment while constantly tracking its own position in the environment as well as the location of the mother, as if the mother serves as a “moving home base" [31]. In this case, the mobility of the "home base" increases the complexity of exploration, now requiring a consideration of both the physical and the social environments. Similarly, proximity to or distance from a high-ranking conspecific could have a major impact on the spatial behavior of other individuals. Indeed, the complexity of a system is determined by the amount of information needed in order to describe it and its level of details [32]. Spatial information, and thereby spatial representation, could become secondary to the social environment: although the individual may need to acquire spatial representation when traveling, it may first choose either to follow or to avoid a companion. Indeed, it was suggested that vervet monkeys are more aware of their social than their physical environment [33]. Similarly, in the present study, the presence of another rat was expected to increase the complexity of spatial behavior of the focal rat, which now had to organize its behavior in relation to both the physical and the social environment. As a result of increased complexity, the structured behavior of individual rats [30,34,35] may degrade, becoming less predictable and more chaotic. In order to uncover such changes in spatial behavior, the present study compared spatial behavior of rats when tested alone and when tested in the same environment together with a companion.

Repeated visits to a specific location, or returning to past places, are an integral part of spatial behavior. Exploration, which is the process of acquiring spatial information on an unfamiliar environment, involves frequent revisits to past places, perhaps to reinforce memory of the locations and for reorientation. Indeed, an earlier study in rats showed that the incidence of revisits decreased with time, when familiarity with the environment increased [36]. Another example of the importance of revisits is path integration, a basic mechanism that enables navigators to take shortcuts regardless of external environmental cues, and in which revisits to the origin of the path increase the accuracy of travel [37–39]. Moreover, in rats, the incidence of revisits to a specific location reflects its importance [9,40], with the home-base location featuring the highest number of revisits [30,41]. Like rats, humans and other animals also organize their behavior in relation to their home site, which serves as a terminal for their spatial behavior [42]. Revisits are thus an important component of spatial behavior. In contrast with the aforementioned biological perspective of "revisits", many paradigms in psychology consider a revisit to a location as an "error". One example of this is the radial maze, comprising several arms (usually 8 or more) with all arms connected together at one end and baited with food at their other, free ends [43]. In such studies, a subject (usually a rodent) is required to collect the food from the arms, ideally without revisiting any arm from which the food has already been collected. Accordingly, a repeated visit to an 'empty' arm is considered an “error”, reflecting a poor working (‘short-term’) memory. This is, however, in contrast with the notion that revisits to past places are an integral part of spatial behavior. These two conflicting notions were examined in the present study, in which rats were challenged by the need to collect food in conjunction with the need to organize their behavior in relation to both the physical and the social environment. Specifically, four main questions were posed in the present study: (i) do dyads of food-deprived rats share or compete for food; (ii) do dyad members dichotomize into a leader ("producer") and a follower ("scrounger") when seeking food; (iii) does spatial behavior become more chaotic and less predictable in the presence of a conspecific; and (iv) when and why do rats tend to keep revisiting objects?

## Methods

### Ethical note

This study was carried out in strict accordance with the recommendations in the Guide for the Care and Use of the Committee on the Ethics of Animal Experiments of Tel-Aviv University (Permit Number: L-14-051).

### Animals

Twenty-four male Sprague–Dawley rats (age 3–4 months; weight 300–400 g) were housed in a temperature-controlled room (22 ± 1°C) under an inverted 12/12-h light/dark cycle (dark phase 8:00–20:00). Rats were held in standard rodent cages (40 x 25 x 20 cm; two rats per cage) with sawdust bedding and were provided with ad-libitum access to water and standard rodent chow. For each cage, rats were marked with a waterproof marker on their tail, one rat with a single stripe and the other with a double stripe.

### Apparatus

**Fig 1:**
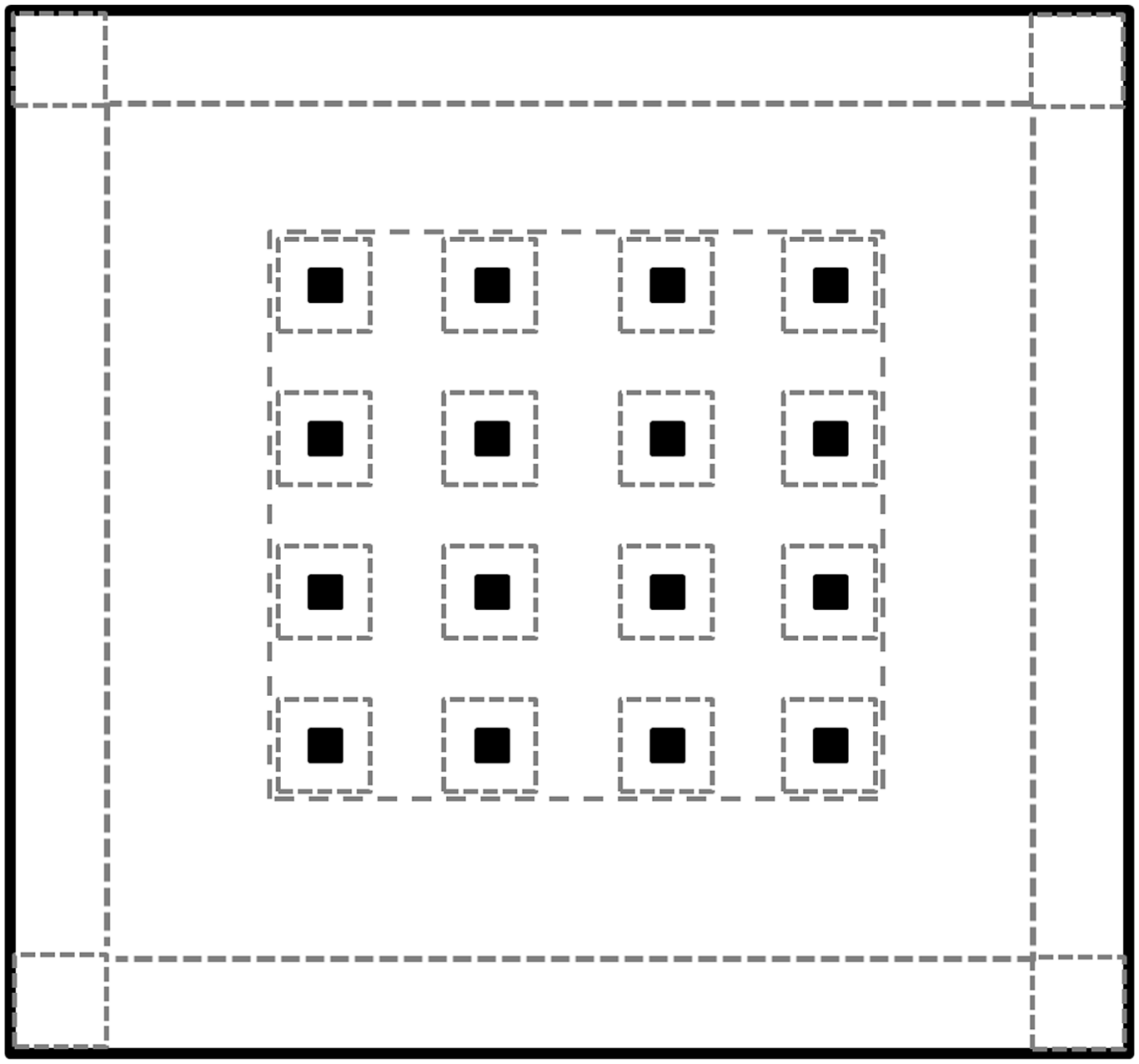
A plot of the arena, objects, and zones. The large circumference represents the 6 x 5.6 m arena, and the 16 dark squares (■) represent the 16 equispaced objects. Dashed lines represent the virtual division of the arena as used by the tracking system. The dashed square around each object represents the object zone, and the 16 object zones together comprise the grid zone. Four corner zones and four perimeter zones (each connecting two adjacent corners) were determined along the walls of the arena.

Rats were tested in a 6 x 5.6 m arena, comprising the white floor of a light-proofed air-conditioned room (22 ± 1°C). The room was illuminated with four cool-white LED projectors (8W each), sufficient to distinguish between subjects but still subtle enough to prevent discomfort to the rats. Sixteen objects (each a 12 x 12 x 6 cm cement cube) were placed in a grid layout, equispaced at 90 cm from each other in the center of the arena (see Fig 1). These objects stood up against the background of the floor, landmarking the location of food items. Trials were recorded by four equispaced Mintron MTV-73S85H color CCTV cameras, placed 2.5 m above the arena, each providing a top view of one of the arena quarters. The four video images were integrated and tracked as one image by a tracking system (Ethovision 10; Noldus Information Technologies, NL) at a rate of five frames per second.

### Procedure

Training and testing were carried out during the dark phase of the rats’ dark/light cycle. The rationale was to test the rats during the period when they are more active. Each rat underwent a series of 40-min training sessions preceded by 12 hrs of food deprivation with access only to water. Fifteen minutes before each session, rats were brought to a room adjacent to the apparatus and their backs were gently painted in blue or red waterproof marker, enabling the tracking system to differentiate between them. Each of the 16 objects was then baited with a small piece of chocolate-flavored cereal, which was placed in the middle of the top surface of each object. An individual rat was then placed gently in the near right corner of the arena, and the experimenter left the arena. Training sessions continued until each rat had collected food from at least 15 objects in less than 15 min. Each rat underwent a different number of training sessions depending on their learning rate (mean of 3 sessions, and min-max of 2–6 sessions, respectively). Once both rats in each cage had completed the training sessions, they underwent the test, which consisted of two 15-min trials with baited objects: (i) a ‘Lone trial’, in which each of the two rats was tested alone (the cage-mates were tested one after the other); and (ii) a ‘Dyad trial’, in which both cage-mates were introduced together into the arena. In this procedure, each rat first established its own spatial map of the test arena before the effect of a partner was assessed. We favored to use cage-mates and not unfamiliar partners to reduce the possibility of aggression that could divert the rats from the spatial task. At the end of each trial, the rats were returned to their cages and the arena was mopped with soap and water in order to neutralize odors prior to the next session.

### Data acquisition and analysis

The image of the arena on the computer screen was divided as follows: (i) 'corners'–four 0.4 x 0.4 m areas at the arena corners; (ii) 'perimeter'–four 0.4 x 5 m ca. strips along the walls between two adjacent corners; (iii) 16 0.4 x 0.4 m zones, each containing one object; (v) a 'grid zone'–encompassing all 16 object zones together, including the spaces between them. Behavioral analysis focused mainly on the grid zone, with some of the parameters also referring to the other arenas (Fig 1). The raw dataset, as extracted from Ethovision, are available in S1 and S2 Tables (lone trials and dyad trials, respectively).

#### Parameters of locomotor behavior and coupling between the rats

For the lone and dyad trials, the following parameters were extracted from ‘Ethovision’ for further analysis with Microsoft Excel 2010, R i386 3.1.2 and MATLAB R2013a. 'Distance traveled' is the cumulative metric distance traveled by a rat. 'Time in a zone' was the cumulative time (min) spent by a rat in each zone. 'Visits to a zone' was the incidence of entries to a zone. 'Time at the home base', was calculated by normalizing the time spent in each zone according to the zone size and ranking the zones from high to low. The zone in which the rat spent most of the 15 min of the trial was considered as the ‘home base [30], and this parameter represented the time spent at the home base (min). 'Visits to the home base' was the incidence of entries to the home-base zone. 'Latency to the next object' was the time of first arrival at each of the 16 objects until all objects had been visited or 15 minutes had elapsed. Potential arrival time at unvisited objects was set to 15 min. under the assumption that had these objects been visited, it would have happened after the trial had ended (15 min or more). 'Trips to the grid zone' was the incidence of entries into the grid zone, regardless of the objects visited during the trip. 'Coupled trips to the grid zone' was a sub-group of trips to the grid zone in the dyad trials that comprised trips in which both rats entered the grid zone and stayed there together for a certain overlapping time-interval. 'Shared objects per coupled trip' was the number of objects visited by both rats in the course of the same coupled trip. 'Total number of object visits' was the cumulative number of visits to objects in the course of the entire trial (repetitions included). 'Visited objects per trip' was the average number of visited objects per trip to the grid zone (repetitions included). 'Trip duration' was the average duration of trips to the grid zone, and it was calculated without distinguishing between coupled and uncoupled trips.

#### Recurrence Quantification Analysis (RQA)

**Fig 2:**
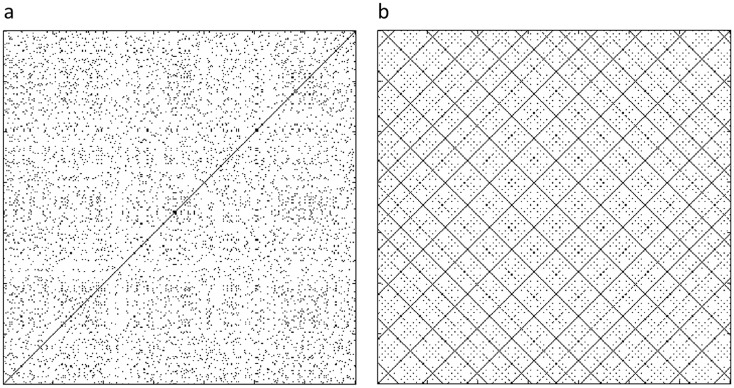
An hypothetical example of recurrence plots. A completely chaotic signal of white Gaussian noise, 350 steps of power 0 dBW (a) generates no diagonal lines at all or very short diagonal lines and stochastic signals. In contrast, a completely periodic signal of a sine wave, also 350 steps (b) generates very long diagonal lines.

Recurrence quantification analysis (RQA) was performed to further explore the rats’ spatial dynamics. RQA is a non-linear analytic method which captures dynamic properties that are lost by averaging in standard correlational methods [44–47]. For the RQA analysis, time series were generated for each rat by measuring its distance from a fixed **focal point** (arena center) at a rate of five frames per second. To reveal patterns in spatial behavior, a recurrence plot was generated for each rat by plotting its time series along both the abscissa and the ordinate. Two recurrence plots were thus generated for each rat: one for the lone trial, and the other for the dyad trial (Fig 2).

The following parameters were extracted from the recurrence plots (see Fig 2). 'Determinism' measures the proportion of recurrent points that form diagonal-line structures. These structures are related to the predictability of the system. In the present study, diagonal line segments had a minimum length of two samples (0.2 sec each). The term determinism represents repetitive or deterministic patterns in spatial behavior, as represented by the time series. 'Diagonal line length' is the average length of the diagonal lines in a recurrence plot. In terms of behavior, a diagonal line meant that the rat was repeating a specific sequence of distances from the center during that trial. Short diagonal lines indicated that the recurrence was more chaotic, while long diagonal lines indicated a more periodic recurrence. The absence of diagonal lines indicated a random recurrence. 'Laminarity' measures the proportion of recurrent points that form vertical-line structures. These structures reflect stationary states of the system. In the present study, vertical line segments had a minimum length of two samples (0.2 sec each). 'Trapping time' is the average length of a vertical line structure. Longer vertical lines represented a tendency to stay at the same distance from the center without progressing.

#### Sample cross-correlations

Sample cross-correlations between the momentary (every 0.2 sec) distance from the focal point in the lone and the dyad trials were calculated. For each pair of rats, two cross-correlations were performed, comprising one rat against its partner: (i) in the dyad trial; and (ii) in the lone trial (hypothetical correlation as the two rats were each tested alone). Maximal correlation of the two rats in the dyad trial was extracted and the lag between the samples of each two rats was calculated.

### Statistics

#### Parameters of locomotor behavior and coupling between the rats

Behavior of the same rats in the dyad and lone trials was compared by means of paired t-tests, unless the data diverted from normal distribution; in these cases a Wilcoxon signed rank-test was performed. For the dyad trial, the average number of objects shared by both rats was compared with the average number of unshared objects for each rat by means of one-way ANOVA with repeated measures. Latency to travel to the next object was compared between the lone and dyad trials by means of two-way RM-ANOVA.

#### Recurrence Quantification Analysis (RQA)

The four parameters of the recurrence plots for the lone and dyad trials were compared by multivariate analysis of variance (MANOVA).

#### Sample cross-correlations

A paired t-test was performed on the transformed correlation coefficients (Fisher transformation) in order to reveal whether the rats' travel was significantly more correlated in the dyad compared with the lone trial. The lag between the two rats in the dyad trial was compared to a hypothetical no lag situation (lag = 0) by means of a t-test. In all tests we used two-tailed hypotheses (where applicable). In all tests alpha level was set to 0.05.

## Results

### Rats performed better when alone than when in dyads

**Fig 3:**
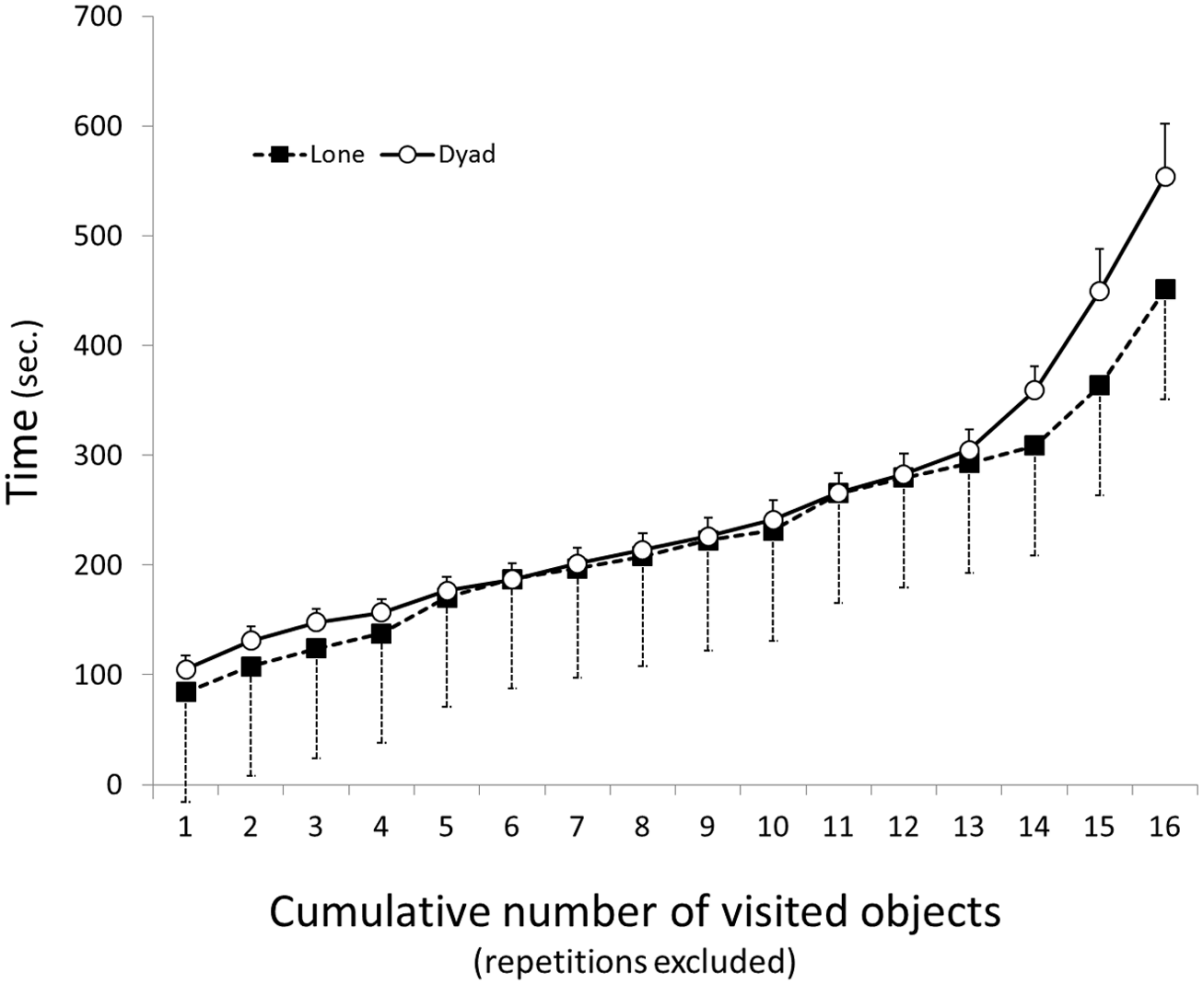
Latency (mean ± SEM) of arrival at each of the 16 objects. The cumulative number of visited objects is depicted along the abscissa, and the time (sec.) of arrival along the ordinate. As shown, the first arrival at each of the 16 objects consistently occurred earlier in lone compared with dyad rats. The short error bars in dyad rats represent the coupling in arrival time, when rats followed one another. In contrast, the greater variance in the lone trial represents the independence of arrival when rats were tested alone.

As detailed in the ‘Methods’, each of the 24 rats was tested twice, first alone and then in a dyad. The following results are based on comparing the behavior of the 24 rats when tested alone with their behavior when tested in dyads. The rats were food-deprived 12 hrs before each trial. During the trial they had access to 16 objects, each baited with a piece of chocolate-flavored cereal. To access the food, the rats traveled from the perimeter of the arena to the grid zone, and during each such trip they visited several objects to collect food. Nevertheless, most objects were visited more than once throughout the trial. During the 15 min lone trial, 19 rats collected all 16 baits, three rats collected 15 baits, one rat collected 13 baits, and one rat collected 9 baits only. During the 15 min of the dyad trial, 19 rats collected all 16 baits, four rats collected only 15 baits, and one rat collected only 14 baits. In terms of task completion, (collecting all baits) rats performed approximately equally in the lone and dyad trials. A two-way RM-ANOVA revealed a significant difference in the latency to the first visit to each object (F_15,268_ = 23.571; p < 0.001). This result is, however, the outcome of ordering the objects from first visited to last. There was a significant difference between the lone and the dyad trials (F_1,368_ = 9.117; p = 0.003), implying that the latency to the first visit to each object was longer in the dyad than in the lone trial (Fig 3). Indeed, the latency in lone trials was consistently shorter than in the dyad trials. The interaction between trial and latency to the first visit to each object was not significant (F_15,368_ = 0.999; p = 0.455).

**Fig 4:**
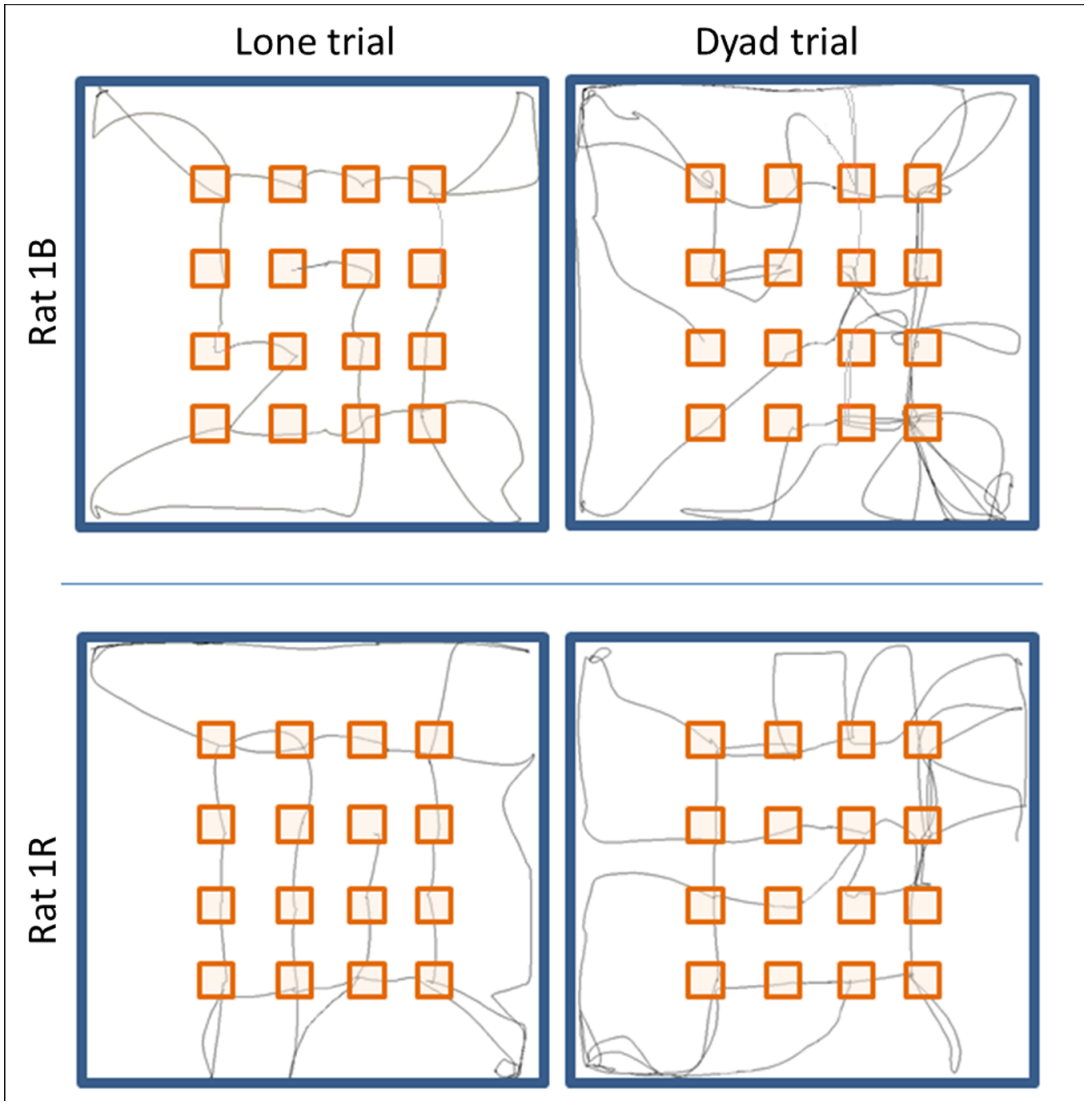
Trajectories of two exemplary rats of the same dyad. The route of each rat in the lone trial is shown in left and the rout of each rat in the dyad trial is shown in the right. The trajectories are depicted until all objects were visited at least once.

Rats in the dyad trial traveled greater cumulative distance, took more but shorter trips from the perimeter to the grid zone, and paid more visits to the objects (repetitive visits included), but these were paid to fewer objects in each trip (Table 1). It should be noted that in both the lone and dyad trials, rats made three to four repeated visits to each object, accumulating more than the 16 visits needed to collect all the baits (see row 3 in Table 1). This implies that collecting food was not the sole aim of the visits. Overall, when tested alone, rats seemed to be more efficient: despite completing the task within approximately the same duration as when tested in dyads, they traveled a shorter cumulative distance and took fewer trips to the grid zone due to visiting more objects during each trip. Consequently, they spent more cumulative time and paid fewer visits to the home base. In contrast, rats in dyads took many short trips to the grid zone, and traveled back and forth between the perimeter and the grid zone, traveling a greater distance but visiting fewer objects per trip. Consequently, they spent less cumulative time and paid more visits to the home base (Table 1). This pattern of behavior is shown in Fig 4 for two exemplary rats.

**Table 1:** Behavior of rats in the lone and dyad trials. Mean (± SEM) of parameters that depict the behavior during the 15 min trials.

Parameter	Lone individuals	Individuals in a dyad	w_23 /_ t_23/_ f_23_	P (two tailed)
Cumulative distance (m.)	158.133 ± 5.785	190.058 ± 4.462	w = -4.286_a_	< 0.0001
Total number of trips to grid zone	11.130 ± 0.490	15.630 ± 0.645	w = -3.934_a_	< 0.0001
Total number of visits to the objects	51.250 ± 2.791	58.208 ± 2.134	w = -2.544_a_	0.011
Trip duration (min.)	0.585 ± 2.791	0.430 ± 0.020	w = -3.486_b_	< 0.0001
Mean number of visits per trip	4.656 ± 0.218	3.771 ± 0.099	w = 2.069	< 0.001
Time in the home corner	22.736 ± 2.508	17.403 ± 1.044	t = 2.069	0.040
Visits to the home base	6.750 ± 0.457	11.792 ± 0.811	t = 2.069	< 0.001

Trials were compared by a Wilcoxon Signed Rank test: a—based on negative ranks; b—based on positive ranks. A paired sample t- test was performed on the difference between the average number of visits per trip of the rats when tested with a partner compared with being tested alone. Test parameters are depicted in the table above. The differences are normally distributed (Shapiro—Wilks normality test results: w = 0.975; p value = 0.7976).

### Spatial behavior of rats in dyads was coupled

In order to reveal whether spatial behavior of rats in dyads is coupled, we performed a sample cross-correlation between the momentary distances (at 0.2 sec intervals) from the focal point of the arena of each of the 12 pairs of rats. Specifically, for each dyad the sample cross-correlation was first performed for the two rats when tested independently in the lone trial, and then when tested together in the dyad trial. The set of correlation coefficients showed that the correlation among distances from the focal point of the arena of two rats in the dyad trial (0.69 ± 0.04; mean ± SEM) was greater than that of the same individuals in the lone trial (0.10 ± 0.04; mean ± SEM). Indeed, a paired two-tailed t-test between the correlation coefficients revealed a significant difference (*t*_11_ = 10.98, p < 0.0001). In other words, the two partners of a dyad kept at the same time the same distance from the focal point, significantly more times than in their lone trial. This implies that spatial behavior of the dyad rats was affected by the partner and was not a mere product of the endogenous and/or physical environment: that is, the behavior of rats in a dyad was coupled in terms of remaining at the same distance from the focal point at the same time. Since the grid zone was located in the central region of the arena, this also implies that the dyad rats synchronized their trips from the perimeter to the grid zone and back. Nevertheless, the rats could have occupied different locations that were equidistant from the focal point. Below we show that this was not the case and that the rats tended to follow one another, and, consequently, to be at the same time in near to one another at the same distance from the focal point.

**Fig 5:**
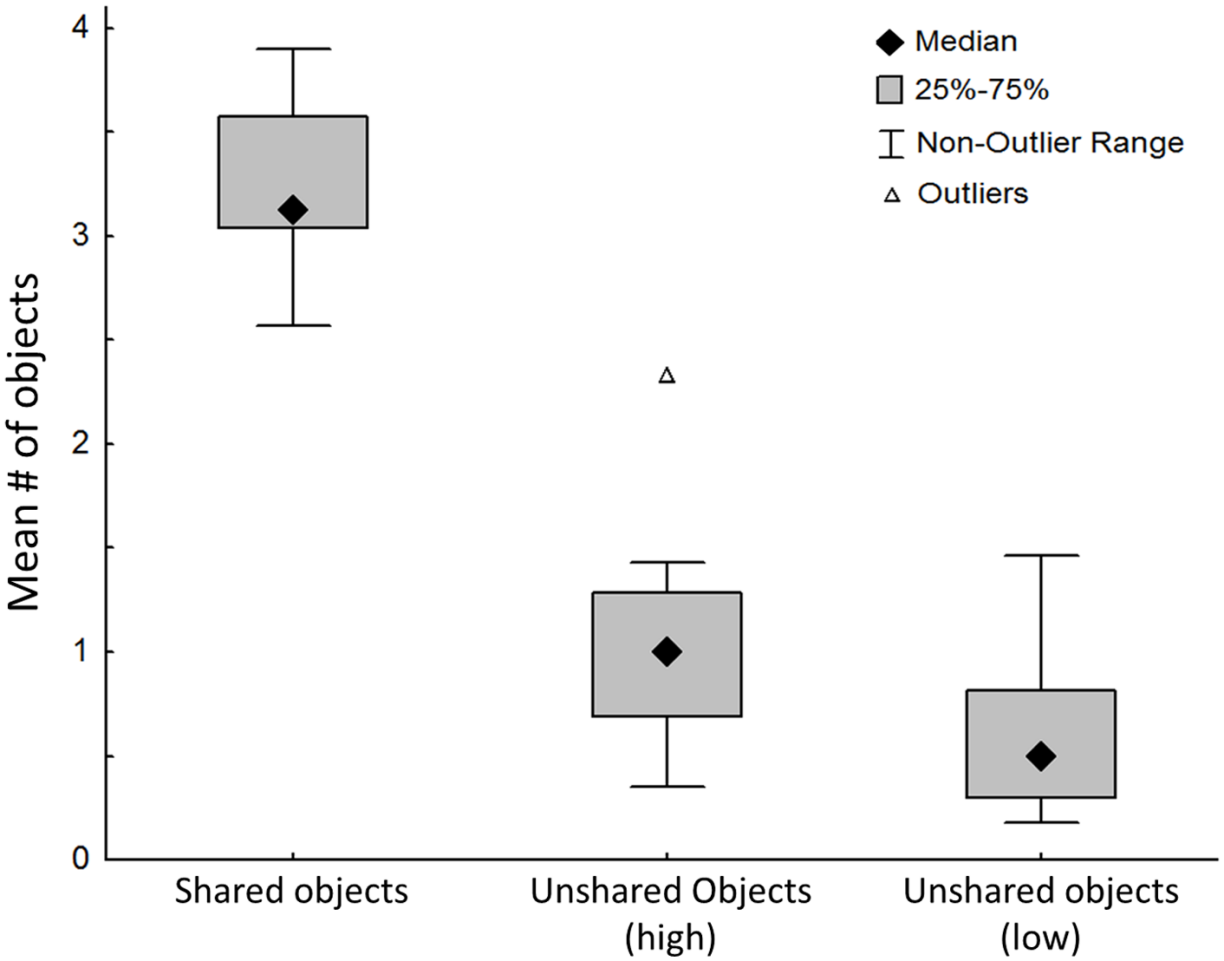
Shared and unshared objects in the dyad trial. For each rat, we extracted the number of objects visited in each trip to the grid zone, and classified the objects into those shared with the other rat, and those unshared with it. For each dyad, three means were extracted: (i) 'shared objects'–average number of objects visited by both rats on the same trip; (ii) 'unshared objects (high)'–average number of objects visited by the rat with the higher number of unshared objects per trip; (iii) 'unshared objects (low)'–average number of objects visited by the rat with the lower number of unshared objects per trip. The median of the 12 dyads (◆) is depicted with the 25% and 75% quintiles (grey square), along with the non-outlier range and the outliers. As shown, both rats in the 12 dyads had significantly more shared than unshared objects.

To collect the food, the rats performed several trips from the perimeter into the grid zone, visiting several objects in each such trip (Fig 4). Trips in which both rats stayed at the same time in the grid zone were termed coupled trips (see 'Methods' section). There were 13.25 ± 2.45 coupled trips *vs*. 5.542 ± 0.606 uncoupled trips per dyad; w = 2, p < 0.0001, in two-tailed Wilcoxon test). The rats also tended to visit a similar set of objects in each coupled trip. Specifically, in 80% of the coupled trips there were 1–5 shared objects, in 16% of coupled trips both rats visited the same 6–10 objects; and in only 4% of the coupled trips did the two rats visit completely different sets of objects. Indeed, as shown in Fig 5, there were significantly more shared than unshared objects per coupled trip. This result illustrates the coupling between rats in dyads, which is also shown in the S1 Videoclip of an exemplary coupled trip. Notably, the coupling of spatial behavior hindered the efficiency of completion of the task in dyads compared with the performance of the same rats in the lone trials (Table 1).

### Rats in dyads were categorized as leaders or followers

In order to determine whether one of the rats in the dyad was leading the other, we compared the maximal correlation of the two rats in the dyad trial, and found that there was a lag of 3.75 ± 0.71 samples (mean ± SEM) between them (sample = 0.2 sec), indicating that one individual was lagging behind the other with respect to the focal point. To reveal whether there was a meaningful trend of one rat leading and the other trailing, we compared the lag to a hypothetical situation in which there was no leader or follower (lag = 0). Indeed, the lag between rats in a dyad was significantly greater compared to the hypothetical no-lead dyad (t-test, *t*_11_ = 3.747; p = 0.0032). Therefore, we interpret this as an indication that in the dyad trial, one rat was a leader and the other was a follower. When traveling in the grid zone, the leaders were first to arrive at the majority of baited objects (11.1 ± 0.9 and 4.8 ± 0.9 first arrivals at baited objects by leaders and follower, respectively; W = 3.5, p = 0.014, Wilcoxon signed rank test).

### Behavior of rats in a dyad is less structured than when alone

**Fig 6:**
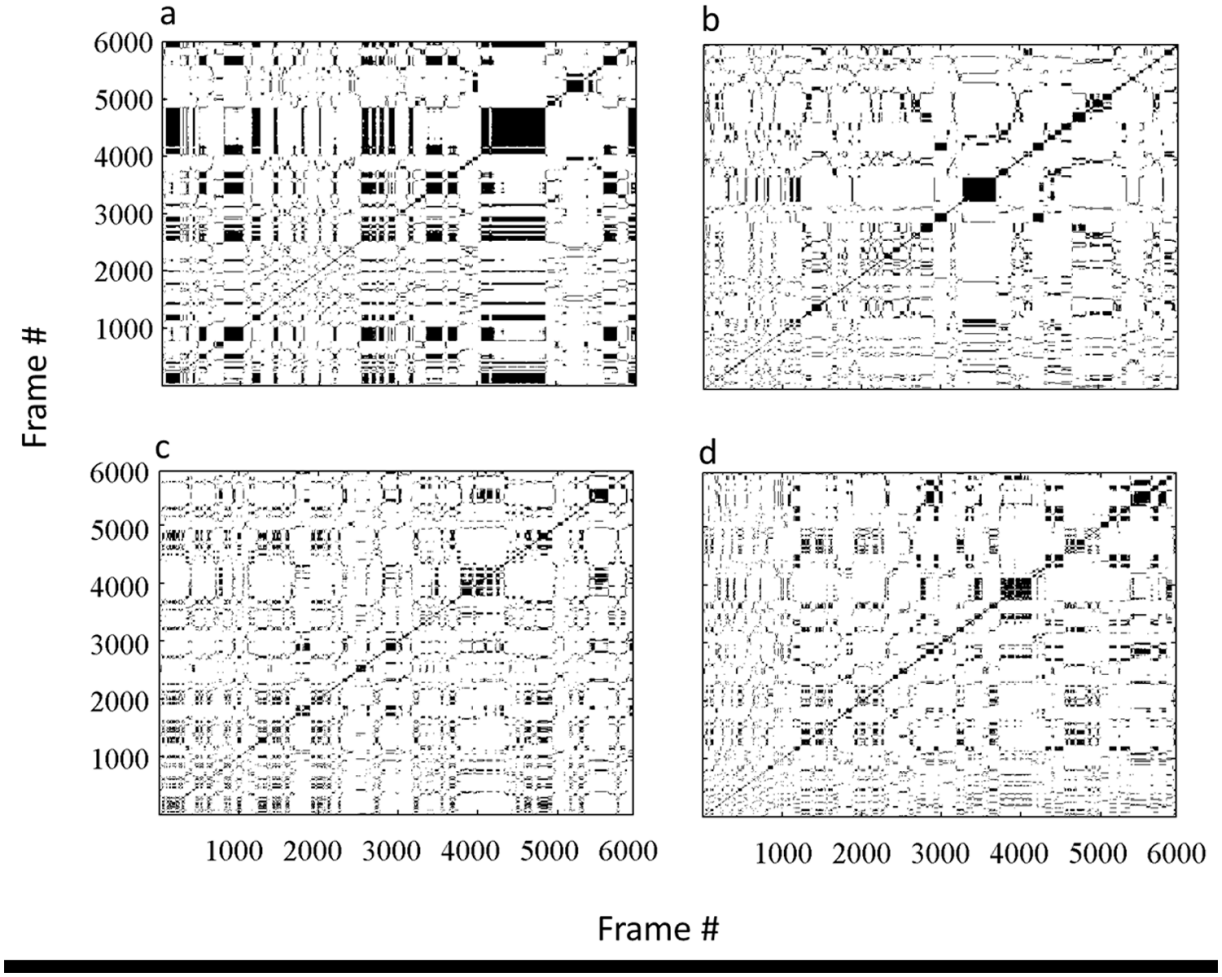
Recurrence plots of two rat of the same dyad. Plots a,b represent, respectively, rat 1 when tested alone and when tested in a dyad. Similarly, plots c,d represent rats 2. When tested alone (left insets) the rats exhibited more periodic and predictable behavior, as reflected in the long vertical and diagonal lines that result in densely packed rectangles, which indicate frequent returning to locations at the same distance from the focal point. In contrast, when the same rats were tested in a dyad, the recurrence plot features smaller squares and shorter diagonal and vertical lines (right insets). These indicate less frequent returning to locations at the same distance from the focal point, and that the rat stopped for shorter periods of time when repeatedly visiting a location. Altogether, the behavior of the rats in the dyad trial (b,d) was more chaotic and less predictable compared to their behavior in the lone trials (a,c).

As described in the ‘Methods’ section, recurrence quantification analysis (RQA) was performed to further explore the rats’ spatial dynamics. For this analysis, the rat’s distance from the focal point of the center arena, sampled at a rate of 5 times per second, was plotted along both the abscissa and the ordinate, and examined qualitatively. This was performed for each rat twice: first as a lone individual and then as an individual in a dyad (Fig 6; see ‘Methods‘ and Fig 2). These two-dimensional plots, which visualize recurrences in time series, revealed different patterns of spatial organization between the behaviors of the same rat in the lone compared with the dyad trial. As illustrated in Fig 6, the plot of the rat in the lone trial was densely packed with relatively longer diagonal and vertical lines of recurrence, resulting in larger rectangular structures, indicating that the system is temporarily paused. In terms of behavior, a diagonal line means that the rat is repeating a specific sequence of distances from the focal point. Short diagonal lines indicate that the recurrence is more chaotic, while long diagonal lines indicate more periodic recurrence. The absence of diagonal lines indicates random recurrence. Longer vertical lines represent a tendency to stay at the same distance from the focal point without progressing. In the plot, long vertical and diagonal lines together result in rectangles, indicating that the rat stayed for a while at the same distance from the focal point. The dense packing of rectangles indicates frequent return to locations at the same distance from the focal point. In contrast, in the plot of the same rat for the dyad trial, recurrence patterns exhibit less deterministic and more chaotic spatial dynamics: the smaller squares and shorter diagonal and vertical lines indicate that the rat stopped for shorter periods of time and, when repeatedly visiting a location, stayed there for shorter periods of time.

By applying this analysis to all rats in both lone and dyad trials, we extracted the variables depicted in Table 2. These variables, derived from the data on the momentary distance from the focal point of the arena, were compared in a one-way multivariate analysis of variance (MANOVA) for the 24 rats in the two trials. Based on Pillai’s trace, the analysis showed a significant main effect of the trial: *V* = 0.55, *F*_4,19_ = 12.99, *p* < 0.0001, across all four variables. As shown, all variables were significantly greater when rats were tested alone compared with when tested in a dyad. Specifically, the greater 'determinism' (the predictability of the plot as the percentage of recurrent points that form diagonal lines) in lone compared with dyad rats demonstrates their overall tendency to maintain longer sequences. A sequence of distances from the focal point represents a rat returning to the same locations, moving at the same distance from the focal point, or simply staying at the same location. The 'averaged diagonal length' shows that repeated bouts of distances were longer on average in the lone trial than in the dyad trial. This implies that locomotion of rats in the dyad trials was more interrupted compared with their lone trials. The greater 'laminarity' (the smoothness of the plot as the proportion of recurrent points that form vertical lines) in lone compared with dyad rats demonstrates their overall tendency to remain at the same distance from the focal point. The 'trapping time' (the average vertical line length), shows that their average time of remaining at the same distance from the focal point was longer when alone compared with when tested in dyads. This parameter provides another representation of the greater number and shorter duration of trips to the grid zone in the dyad compared to the lone trials (see Table 1).

**Table 2:** Recurrence variables. The mean (± SEM) for each variable is depicted for lone individuals and for individuals in a dyad, along with the results of one-way multivariate analysis of variance (MANOVA) for the two conditions (individuals/dyads).

Parameter	Lone individuals	Individuals in a dyad	F_4,19_	p
Determinism	0.984 ± 0.002	0.965 ± 0.002	51.52	< 0.0001
Averaged diagonal length	15.391 ± 1.671	7.352 ± 0.347	22.19	< 0.0001
Laminarity	0.985 ± 0.002	0.967 ± 0.002	41.58	< 0.0001
Trapping time	22.683 ± 2.597	9.979 ± 0.557	22.87	< 0.0001

Altogether, the behavior of lone rats was more organized in time and space, perhaps since they were organizing their behavior only in reference to the physical environment. In contrast, the behavior of rats in dyads was more chaotic, perhaps since they were organizing their spatial behavior in reference to both the physical environment and their partner. In other words, spatial behavior of lone rats was more structured (less chaotic, or more predictable) than their behavior when part of a dyad.

## Discussion

In the present study, food-deprived rats were trained to collect a small item of food from 16 equispaced objects in a large arena. They were then tested in the same arena with another food-deprived rat (their cage-mate) which had also been trained in the same procedure. The results revealed that each of the rats performed more efficiently when tested alone than when tested in a dyad, in terms of latency to collect all the baits. Moreover, spatial behavior in dyad rats was coupled: they traveled together while visiting the same objects, with one partner seemingly leading the other. Finally, recurrence quantification analysis revealed the behavior of rats in a dyad to be less structured than when alone. In the following discussion, we first suggest that even for food-deprived rats, foraging does not necessarily predominate and spatial behavior is also shaped by other factors. We then discuss the tendency of one rat to follow its mate, and suggest that foraging and consuming food together is adaptive in rats. We also explain why behavior in dyads is more chaotic and less predictable, and ultimately call for caution when interpreting revisits as an error in spatial tasks.

### Spatial behavior of a dyad of food-deprived rats cannot be explained only in terms of foraging

Our results revealed that compared with their behavior in the lone trials, rats in dyad trials traveled greater distances, spent less time in the home corner, paid more visits to the home base, made more trips to the grid zone, and paid more visits to the objects, but visited fewer objects per trip (Table 1). In the dyad trials, both partners tended to visit the same set of objects in the same trip. In other words, rats tended to remain in the vicinity of their partners, at least during trips to the grid zone. Since only one small piece of bait was placed on each object, only 16 visits (one per object) were necessary in order to acquire all the food. However, rats in both the dyad and lone trials paid much more visits than were necessary, and performed repeated visits to most objects (Table 1). Implicit in the repeated visits is that foraging was not the only driving force in the spatial behavior of the dyads. Other factors, such as memorizing paths or returning to the safety rendered by specific locations (familiar places, the arena walls and corners) were probably involved in shaping the behavior of the rats and diverted them from optimal foraging behavior despite food deprivation [48]. In other words, there was more to navigating space to find food than the food itself. The diversion from foraging was especially notable in the presence of a partner, when the rats seemed more focused on one another than on foraging.

### Leaders and followers

As shown in the present study, one of the rats was leading and the other lagging behind (see S1 Videoclip). The leader was also the first to arrive at twice as many baited objects as the follower. Spatial behavior of the leader and follower in each dyad seemed to be dominated by three major factors: (i) the physical structure of the environment; (ii) the presence of food (16 equispaced baits); and (iii) the presence of another rat. While the last factor strongly influenced the spatial behavior of both dyad members, to the observer it seemed as if followers were affected more by the presence of the partner.

The division into leaders and followers appears to reflect the "Producer—Scrounger model" [49]. This model predicts that animals use two main foraging tactics: finding sources of food on their own (producers); or relying on other feeding animals to locate food (scroungers). Being a producer or a leader may be a profitable trait, as they are the first to arrive at food sources. Being a scrounger or a follower, on the other hand, is easier, as fewer decisions have to be made. Altogether, the present results indicate that leaders are more independent in their spatial behavior, whereas followers tend to consider more the position of others when deciding where to go next.

### A possible adaptive value of foraging together

Whether the division into leaders and followers in a group of foragers has a certain adaptive value or not, group foraging facilitates the acquisition of public information and thereby increases the chances of group members finding food sources; however, it also increases the competition among individuals over these food sources [50]. Many models of social foraging make predictions about the tendency of animals to forage in groups and about the optimal group size [11]. In this context, wild rats live in colonies, and members of the colony tend to forage in groups [51]. Group foraging involves the transmission and reception of public information that enables individuals to assess the residual value of a patch in light of the success or failure of their group members [52–54]. Indeed, animals are drawn to a location by the mere presence of feeding conspecifics (local enhancement; [55]. This process is particularly important for omnivores, like wild rats, that forage on a wide range of food types, with the potential for costly errors in food selection [56]. Moreover, wild rat populations are exposed to the pesticides that are added to their preferred foods. Accordingly, rats rely on their conspecifics' experience in order to determine what, where, and when to eat [57,58]. In other words, for rats, witnessing the outcome of food consumption by others is vital since even a familiar food could be harmful. Following the eating behavior of others therefore directs them towards wholesome foods and the avoidance of harmful ones. This could be a more critical than locating food efficiently, and this behavioral trait appears to be retained even by laboratory rats, which display group foraging when transferred to natural settings [29]. The present finding that rats tend to follow their partner when collecting food may thus be interpreted as an exhibition of the ancestral adaptive need to follow the eating behavior of others. Accordingly, it could be that for rats in the present study it felt safer, and in a sense easier, to follow their partners at the expense of efficiency.

### Behavioral facilitation in dyads

As mentioned before, foraging was not the only driving force in structuring spatial behavior since the rats continued to make trips to the grid zone and to visit objects even after all the objects no longer contained food. In the dyad trials, foraging seemed to be an even less meaningful vector in shaping spatial behavior since the food-deprived rats were more attentive to one another than to the food. Would foraging be the rats' main concern, they should not follow one another but head directly to the food items, ideally visiting different set of objects than those visited by their partner. Clearly, this was not the case and the rats were mainly following one another. Besides their tendency to remain together, the rats were more active in the dyad trials, compared with their behavior in the same environment in the lone trials (Table 1). This social facilitation of activity was previously found in rat dyads that were tested in an empty open-field, where there was no food to forage for [9]. Accordingly, this behavioral change is merely induced by the presence of another rat. One explanation for this could be an elevated sense of security rendered by the presence of a conspecific [59,60]. Indeed, animals in groups may benefit from an increased probability of detecting a predator [61], and thus be able to reduce individual vigilance without reducing the probability of detecting the predator by the group [62,63]. Therefore, group members can spend more time in foraging as well as in other activities.

In social facilitation by animals in groups (dyads or more), the animals are more active, display lower neophobia, and explore new objects more thoroughly than individuals do [59,64–71]. However, social facilitation has been shown to occur only with well-practiced or simple tasks, whereas with unfamiliar or complicated tasks performance may degrade in the presence of others [72]. Since in the present study each rat was first trained alone, and only then performed the same spatial task alongside another trained rat, both rats should have displayed an enhanced performance of the well-practiced task of collecting the 16 baits, and should have completed the collection of baits sooner than when alone. The results, however, revealed an opposite pattern, and it took the rats more time to visit all 16 objects in the dyad trial. This diversion from the expected pattern of social enhancement is, however, in line with the interpretation that the food-deprived rats were attending more to one another than to the objects and the available food, attesting again to the primacy of socializing over collecting food (performance).

### Higher complexity in the dyad trial

The coupled spatial behavior of the dyad members turned out to be more complex than their spatial behavior when alone. In lone rats, spatial behavior is organized in relation to one or more points of reference, with the 'home base' being the most prominent point [30]. Indeed, 'home' is not a mere physical construct or location, but first and foremost a hub and organizer of spatial behavior [42]. In the present experiment, rats were tested in the same physical environment, first alone and then with their cage-mate. In this procedure, lone rats faced only a spatial task, whereas a social factor was added in the dyad trial. This increased the complexity of the spatial task since the rats were now both occupied with the spatial task and concurrently interacting with their partner (which were also occupied with the same spatial task). In other words, rats in dyads had to organize their spatial behavior in reference to the physical environment as well as to a moving point of reference—the other rat. Moreover, in terms of working memory, the dyad trial was a more challenging task than the lone trial. Working memory refers to short-term memory as it is used to plan and carry out behavior [73]. For example, in order to decide where to go next, a rat needs to know which locations it has already visited. In the dyad trial, a rat needs to take into account the locations it visited along with the locations visited by the other rat. Therefore, the dyad trial included not only an addition of a moving focal component that amplified complexity of the spatiotemporal organization of behavior, but it also comprised a greater challenge for working memory than in the lone trial. Consequently, the combination of social and physical environments resulted in a more chaotic and less predictable spatial behavior in the dyad rats.

### Can revisiting be considered an “error”?

As detailed in the "Introduction", a repeated visit to a maze arm is usually considered an “error” in many psychology paradigms. In a study with two rats in an eight-arm radial maze, it was found that the rats preferred locations recently visited by their partner [74], a result consistent with the findings of the present study. Another study aimed at separating the social preference to travel with a partner from the spatial memory of places already visited by the partner (and thus food-depleted). For this, a caged rat was allowed to observe a model rat visiting four accessible arms of an eight-arm radial-maze. The model rat was then removed and the caged rat, which was then free to explore all maze arms, showed a slight preference to visit arms that had not been visited by the model rat [50]. Taking together the tendency of the rats to follow one another and their lesser tendency to avoid places already visited by the model rat, it is suggested here that the presence of a rat is an attractor that outweighs the desire for food. Indeed, rats have a tendency to follow one another [9]. Moreover, wild rats usually forage in groups [51], display a tendency for food-sharing [10], and favor food that is already being consumed by companions [57]. These findings, and especially the latter, have an apparent adaptive value, as discussed in detail above (section 4.3). Accordingly, the contention that arrival at an unvisited location is a “correct choice” whereas a revisit is an “error” does not necessarily follow the biological and social aspects of rats’ natural behavior.

Another argument against the contention that revisiting past places is an error comes from the role of revisits as a means for memorizing locations and facilitating wayfinding (see "Introduction"). From a broader viewpoint, exploration is a process of spatial learning, and learning is primarily based on repetition, with the refreshment of knowledge based on experience learned from the past (self-learning) and extracted from social interactions (social learning)[75]. Altogether, revisiting past locations is an important component of spatial behavior, and caution is thus required when considering it as an "error". Training and familiarity with the task and apparatus are required before the rats avoid revisiting arms, and only after such training might revisits be considered as errors. Even then, this should be limited to tests with individual animals, since the presence of a companion can overshadow the training for avoidance. In the present study, revisits were not necessarily the sign of a fault in spatial memory, but an indication that other factors, like those described above, were also involved in shaping spatial behavior.

## Conclusions

The behavior of the tested rats underwent a substantial change in the presence of another rat. Specifically, food-deprived rats that were trained to collect food from 16 equispaced objects, diverted from this task mainly to attend their companion, with one of them leading and the other trailing. It is suggested that including a social factor in the spatial task added another level of complexity, resulting in the more chaotic behavior of rats in dyads compared with their lone performance. In consequence, task completion was slower and seemingly less efficient in dyads, and the food-deprived rats displayed greater occupation in following one another rather than in collecting the food items. This alleged inefficiency could be adaptive, since group foraging and consuming food together is a natural way for rats to avoid poisoning. It is also argued that the common contention of considering revisits as an error in spatial tasks does not necessarily follow the biological and social aspects of rats’ natural behavior.

## Supporting Information

S1 Table X,Y,T and zones as extracted from the tracking system for the lone trials. (XLSX)

S2 Table X,Y,T and zones as extracted from the tracking system for the dyad trials. (XLSX)

S1 Videoclip The clip depicts two rats performing a coupled trip to the grid zone during which they stop at the same objects. (MP4)
